# *SPL8* Acts Together with the Brassinosteroid-Signaling Component *BIM1* in Controlling *Arabidopsis thaliana* Male Fertility

**DOI:** 10.3390/plants2030416

**Published:** 2013-06-25

**Authors:** Shuping Xing, Vanessa Quodt, John Chandler, Susanne Höhmann, Rita Berndtgen, Peter Huijser

**Affiliations:** 1 Department of Comparative Development and Genetics, Max Planck Institute for Plant Breeding Research, Cologne 50829, Germany; E-Mails: hoehmann@mpipz.mpg.de (S.H.); berndtge@mpipz.mpg.de (R.B.); 2 Institute of Developmental Biology, Cologne Biocenter, University of Cologne, Cologne 50674, Germany; E-Mail: john.chandler@uni-koeln.de

**Keywords:** anther development, *Arabidopsis thaliana*, *BIM1*, brassinosteroid signaling, male fertility, *SPL8*

## Abstract

The non-miR156 targeted SBP-box gene *SQUAMOSA PROMOTER BINDING PROTEIN-LIKE 8* (*SPL8*), plays an important role in Arabidopsis anther development, where its loss-of-function results in a semi-sterile phenotype. Fully male-sterile plants are obtained when a *spl8* loss-of-function mutation is introduced into a *35S:MIR156* genetic background, thereby revealing functional redundancy between *SPL8* and miR156-targeted SBP-box genes. Here, we show that *BIM1*, a gene encoding a bHLH protein involved in brassinosteroid signaling and embryonic patterning, functions redundantly with *SPL8* in its requirement for male fertility. Although *bim1* single mutants displayed a mild fertility problem due to shortened filaments in some flowers, mutation of *BIM1* significantly enhanced the semi-sterile phenotype of the *spl8* mutant. Expression of both *SPL8* and *BIM1* was detected in overlapping expression domains during early anther developmental stages. Our data suggest that in regulating anther development, *SPL8* and *BIM1* function cooperatively in a common complex or in synergistic pathways. Phylogenetic analysis supports the idea of an evolutionary conserved function for both genes in angiosperm anther development.

## 1. Introduction

In flowering plants, functional development of the male stamen and the female pistil is required for successful sexual reproduction and is affected by many genetic and environmental factors. In the model plant *Arabidopsis thaliana*, a typical flower has six stamens and one pistil, or gynoecium, formed by two carpels. Organ identity determination, initiation, growth and patterning of these reproductive structures have been widely investigated [1,2,3,4]. One of our long-term interests is to understand the molecular mechanisms that underlie the development of the anther, the terminal part of the stamen, which produces the male gametophytes or pollen. According to Sanders *et al.* [5], the Arabidopsis anther developmental process can be divided into 14 histomorphologically defined stages. Stages 1 to 7 are early developmental stages, predominantly confined to sporogenous cell and anther wall formation in the four corners of the anther. The later postmeiotic Stages 8 to 14, comprise pollen maturation and anther structural adaptations for its release at anthesis. 

Many genes are known to be involved in these late anther developmental stages, but relatively few genes are known to act during the earlier stages [6,7], when cell division, commitment and differentiation occur. Among these latter genes, *SPOROCYTELESS*/*NOZZLE* (*SPL*/*NZZ*) has been characterized in Arabidopsis. Mutation of *spl*/*nzz* leads to the formation of anthers without a proper specification of sporogenous cells and thus, the absence of pollen sacs [8,9]. Interestingly, loss-of-function of both *ROXY1* and *ROXY2*, two closely-related CC-type glutaredoxin encoding genes, results in a similar phenotype that mainly affects pollen sac formation in the adaxial anther lobes [10]. Recently, *SQUAMOSA PROMOTER BINDING PROTEIN-LIKE* (*SPL*) genes, encoding plant-specific SBP-domain transcription factors, have also been reported to play an important role in early anther development. Mutation of *SPL8* results in reduced seed set, especially in the first few flowers, where the anthers remain partly or fully devoid of pollen due to an early arrest of sporogenous cell formation in all or some of the anther lobes [11]. A complete loss of pollen production in the anthers of all formed flowers is obtained when the *spl8* mutant is combined with a *35S:MIR156* transgene that is able to down-regulate the expression of a set of miR156-targeted SBP-box genes. Such mutant transgenic plants produce fully male sterile organs with anthers lacking all four pollen sacs [12], thereby resembling the *spl*/*nzz* mutant phenotype.

Brassinosteroids, a class of plant hormones [13], have also recently been reported to control male fertility by regulating the expression of several key genes involved in anther and pollen development, such as *SPL*/*NZZ*, *TDF1*, *AMS*, *AtMYB103* and *MS1* [14]. The basic helix-loop-helix (bHLH) protein BIM1 (BES1-interacting Myc-like1) is a brassinosteroid signaling component involved in regulating BR-induced genes [15] and controls embryo patterning via interaction with the AP2 transcription factors DORNRÖSCHEN (DRN) and DORNRÖSCHEN-LIKE (DRNL) [16]. Here, we describe that *BIM1* and *SPL8* function together to control early anther development. Mutation of *BIM1* in a *spl8* mutant background significantly enhanced the *spl8* semi-sterile phenotype, suggesting that the products of both genes act cooperatively in a common complex or via synergistic pathways to promote Arabidopsis male fertility.

## 2. Results

### 2.1. bim1 Enhances the spl8 Semi-Sterile Phenotype

**Figure 1:**
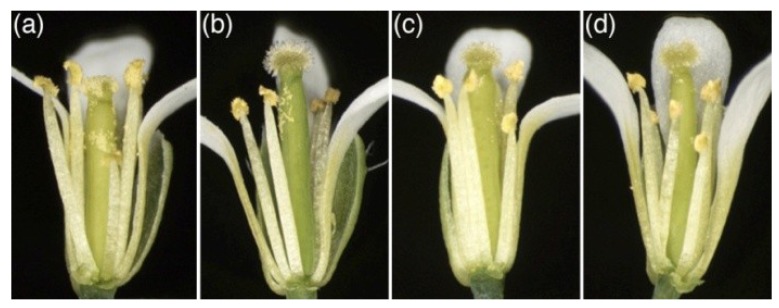
Flower morphology of *bim1* and *spl8* mutants at anthesis. To obtain a better view inside the flower, two sepals and one petal were removed. For comparison, the 10th flower was selected. (**a**) *A. thaliana* Col-0 wild type, showing dehisced anthers with pollen grains and the stigmatic papillae covered with many pollen grains; (**b**) *bim1*-1, the anthers releasing pollen grains onto the surface of the gynoecium valve due to the short filament; (**c**) *spl8*-1, some pollen grains released from anthers touching the stigmatic papillae; (**d**) *spl8*-1 *bim1*-1, a few pollen grains were deposited on the surface of the valve.

**Figure 2:**
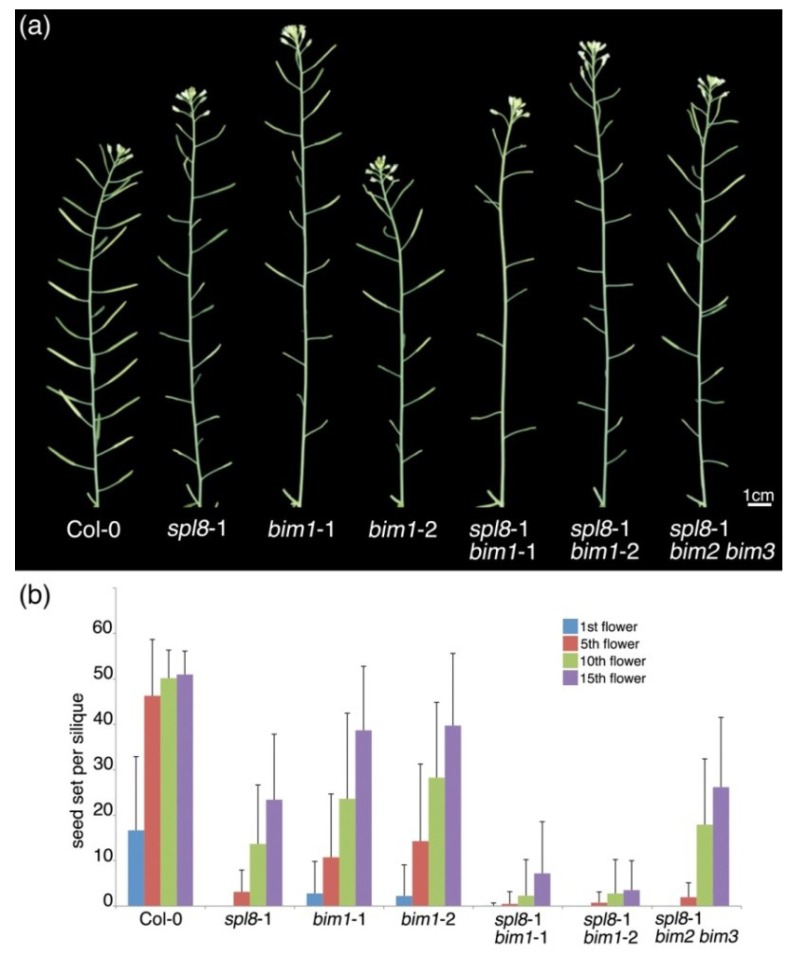
Seed set in *bim*, *spl8* and their combined mutants. (**a**) Primary inflorescences, excised immediately below the last cauline leaf of *A. thaliana* Col-0 wild type and mutant plants as labeled in the image. (**b**) Mean seed set per silique from the first, fifth, 10th and 15th flower, respectively, formed within the primary inflorescences of wild type and mutant plants. Error bars indicate SD (n = 30).

As we have reported previously, the Arabidopsis *spl8* mutant is semi-sterile, and an additional down-regulation of other, miR156-targeted, *SPL* genes results in fully sterile plants [11,12]. In search of additional genetic factors involved in the *spl8* semi-sterile phenotype, we performed crosses between *spl8* and some mutants of candidate genes. This way, we selected the *bim1* mutant for further analysis. Morphologically, the *bim1* single mutant appeared similar to wild type, except that it had a mild fertility problem, probably due to some flowers producing stamens with shortened filaments (Figure 1b). As for *spl8*, the severity of the fertility problem in *bim1* mutant flowers was also found to be dependent on their position in the inflorescence. Whereas the first flowers of the *bim1* mutant primary inflorescence often produced only a few seeds, increasingly more seeds were formed by later-arising flowers (Figure 2a,b). More strikingly, however, seed number was also dramatically reduced in later-arising flowers of *spl8 bim1* double mutants. For example, in the 10th flower of both *spl8*-1 *bim1*-1 and *spl8*-1 *bim1*-2 double mutants, the mean seed number was 2.3 ± 8.0 and 2.8 ± 7.5, respectively, whereas in the single mutants *spl8*-1, *bim1*-1 and *bim1*-2 this mean was 13.7 ± 13.0, 23.6 ± 18.8 and 28.3 ± 16.6, respectively, and 50.2 ± 6.2 in wild type (Figure 2b). Compared to the respective parental mutant lines, however, the length of stamen filaments showed no obvious further reduction in *spl8 bim1* double mutant flowers (Figure 1c,d; five flowers of each genotype were dissected for comparison). The further reduction in fertility of the *spl8 bim1* double mutant could thus not be ascribed to short filaments. We also tested whether mutations in *BIM2* and *BIM3*, both encoding bHLH proteins closely related to *BIM1*, would have an effect on *spl8* fertility. In contrast to *bim1*, however, the *bim2* and *bim3* mutations did not significantly affect *spl8* fertility and seed production of the *spl8*-1 *bim2 bim3* triple mutant remained comparable to that of the *spl8*-1 single mutant (Figure 2b). Likewise, seed production in the quadruple mutant *spl8*-1 *bim1 bim2 bim3* resembled that of the *spl8*-1 *bim1* double mutant (data not shown). These data clearly indicate that *BIM1*, but not *BIM2* or *BIM3*, contribute to the fertility of the *spl8* mutant, suggesting that in regulating male fertility, *BIM1* and *SPL8* act in the same or in parallel pathways. 

### 2.2. Isolation of a New Loss-of-Function Allele for BIM1

**Figure 3:**
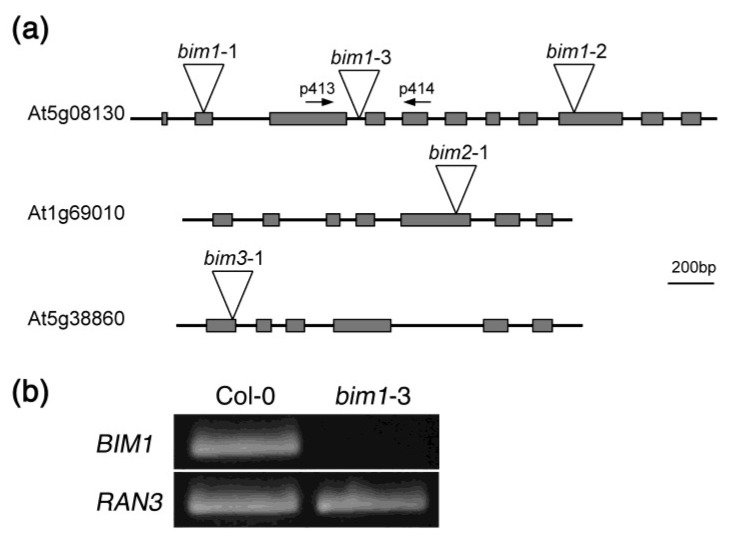
Isolation of *bim1*, *bim2* and *bim3* mutants. (**a**) Structures of *BIM* genes and mutant alleles. The triangle indicates the position of the T-DNA insertion within the gene. P413 and P414 are the two primers used to amplify a portion of the *BIM1* transcript. (**b**) Comparison of *BIM1* expression in inflorescences of *A. thaliana* Col-0 wild type and the *bim1*-3 mutant by RT-PCR. The *RAN3* gene was amplified as a control [10].

To obtain more solid evidence that the enhancement of the *spl8* mutant phenotype in *spl8 bim1* double mutants resulted from the loss of *BIM1* gene function, we isolated another T-DNA insertion mutant allele from the Wisconsin Dslox Project lines (N850504, Nottingham Arabidopsis Stock Centre). The T-DNA was found to be inserted in the third intron of *BIM1*, and we named this new mutant allele *bim1*-3 (Figure 3a). Accordingly, the other two *BIM1* alleles used in this study are referred to as *bim1*-1 (Salk_085924, [15]), *bim1*-2 (Salk_132178, [16]), and those of *BIM2* and *BIM3,* respectively, as *bim2*-1 (Salk_074689, [15]) and *bim3*-1 (Salk_079683, [15]). To test whether *bim1*-3 represented a loss-of-function mutant, we extracted RNA from the homozygous mutant *bim1*-3 and wild type plants and performed reverse transcription polymerase chain reaction (RT-PCR) using a primer pair matching exon sequences flanking the T-DNA insertion site (Figure 3a). This showed that whereas a clear band could be amplified from wild type, no amplicon was present for *bim1*-3, suggesting the latter to be a null allele although it cannot be ruled out that a partial transcript might give rise to residual function. (Figure 3b). Homozygous double mutants generated with this new mutant *bim1*-3 allele and two different mutant *spl8* alleles, *spl8*-1 and *spl8*-3, respectively, showed fertility defects similar to those of the *spl8*-1 *bim1*-1 and *spl8*-1 *bim1*-2 double mutants mentioned above (data not shown). These results further strengthen the conclusion that loss of *BIM1* gene function enhances the *spl8* semi-sterile phenotype.

### 2.3. Histological Analysis of spl8 bim1 Double Mutant Anthers

**Figure 4:**
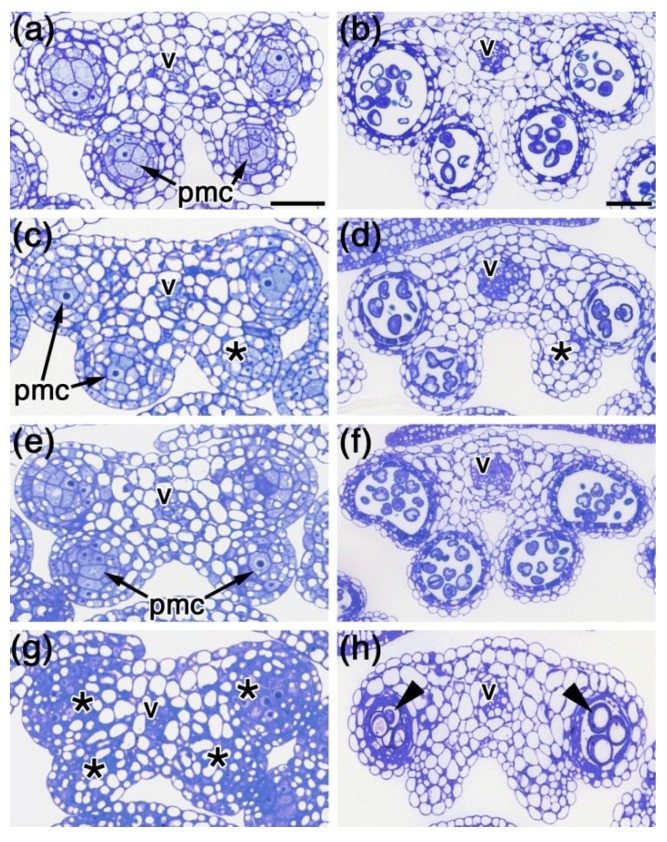
Comparative histological structures of anthers. Semi-thin cross-sections through Arabidopsis wild type (**a**,**b**), *spl8*-1 (**c**,**d**), *bim1*-1 (**e**,**f**) and *spl8*-1 *bim1*-1 (**g**,**h**) anthers from the 10th flower were stained with toluidine blue. (**a**), (**c**), (**e**), (**g**) Anthers at Stage 5, the pollen mother cells (PMCs) are present in the center and are surrounded by distinct anther wall layers in the wild type, *spl8*-1 and *bim1*-1 mutants. Note that the lower right locule of this particular *spl8*-1 anther and all four loculi of *sp8*-1 *bim1*-1 anthers lack typical PMCs (stars). (**b**), (**d**), (**f**), (**h**) Anthers at Stage 8. Pollen grains were formed in pollen sacs of wild type, *spl8*-1 and *bim1*-1 mutants, however, the number of the pollen grains in each pollen sac and the number of pollen sacs was reduced in the *spl8*-1 mutant (**d**), and this phenotype was further enhanced when *spl8* was combined with *bim1*. The abaxial PMCs degenerated and non-functional pollen grains were formed in this particular *spl8*-1 *bim1*-1 anther (**h**). pmc, pollen mother cell; v, vascular bundle. Bar = 50 µm.

As we concluded above, it appeared unlikely that the strong reduction in fertility of the *spl8 bim1* double mutant flowers could be ascribed to the shortened filaments already observed in *bim1* single mutant flowers (Figure 1a–d). As *spl8* single mutant anthers are known to exhibit well-defined developmental defects, we thus focused on the developing double mutant anthers. For proper comparison we always selected the 10th flower from primary inflorescences of wild type, *spl8*-1, *bim1*-1 and *spl8*-1 *bim1*-1 mutant plants, and prepared semi-thin cross-sections from their anthers. At an early anther developmental stage, e.g., Stage 5, wild type showed distinct anther wall cell layers surrounding the pollen mother cells (PMCs) at each of the four loculi (Figure 4a). At a post-meiotic stage, e.g., Stage 8, these loculi had developed into four pollen sacs containing many pollen grains (Figure 4b). Similar developmental stages in both the *spl8*-1 (Figure 4c,d) and *bim1*-1 (Figure 4e,f) single mutant anthers showed a comparable degree of tissue differentiation. Occasionally however, an adaxially-positioned loculus failed to develop in the *spl8*-1 mutant, in which case, sporogenous cells did not appear to be specified (Figure 4c), and thus no functional pollen sac formed (Figure 4d). Clearly, comparably staged *spl8*-1 *bim1*-1 double mutant anthers exhibited more severe developmental anomalies. Sporogenous cell formation appeared to be disturbed (Figure 4g), particularly in the adaxial loculi, resulting in anthers lacking pollen sacs at these positions (Figure 4h). In some cases, the PMCs appeared not to have undergone meiosis and later degenerated in the pollen sacs (Figure 4h). Given these observations, we conclude that *BIM1* and *SPL8* are both required for early anther development.

### 2.4. Expression of BIM1 in Anthers

**Figure 5:**
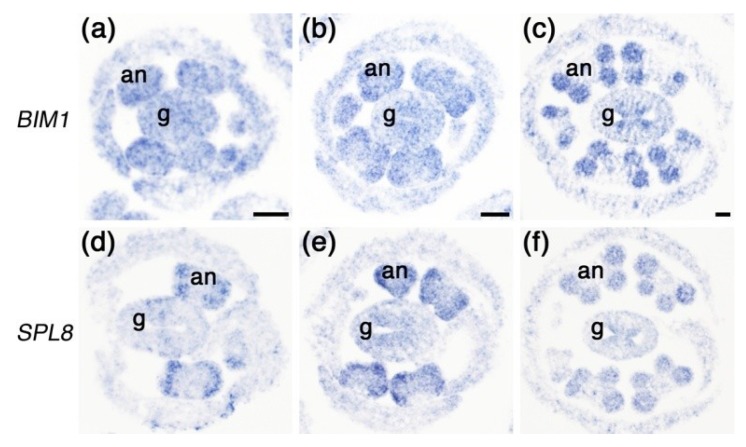
*In situ* hybridization analysis of *BIM1* and *SPL8* in early flower buds from primary inflorescences of Arabidopsis wild type plants. Cross-sections through wild type early flowers at several stages were analyzed for *BIM1* (**a**–**c**) and *SPL8* (**d**–**f**) expression. (**a**,**d**) Cross-sections through the flower buds containing Stage 3 anthers, showing *BIM1* expression in the anthers and gynoecium, and distinct *SPL8* expression mainly in the four corners of the anthers. (**b**,**e**) At anther Stage 4, both *BIM1* and *SPL8* are expressed in all four lobes of the anthers, in both sporogenous cells and somatic cell layers. (**c**,**f**) Before meiosis, at anther Stage 6, expression of both genes is mainly confined to PMCs and anther walls. an, anther; g, gynoecium. Bars = 50 µm.

As the genetic and histological data revealed the function of *BIM1* to be required for proper anther development, we performed *in situ* hybridization experiments to investigate *BIM1* spatial and temporal expression in anthers. For comparison, we hybridized a *SPL8* probe in parallel. *BIM1* expression was already clearly detectable in early Stage 3 anthers, when archesporial cell divisions in the four corners start to give rise to the sporogenous tissue [5]. In this early stage, the *BIM1* hybridization signal spread over the entire anther and gynoecium cross-section (Figure 5a), whereas *SPL8* hybridization appeared only in the four corners of the anthers and was weakly detectable in medial regions of the gynoecium (Figure 5d). At the subsequent developmental Stage 4, when more sporogenous cells had formed, *BIM1* expression started to resemble broadly that of *SPL8*, *i.e*., more confined to the four corners of the anthers and the medial regions of the gynoecium (Figure 5b,e). At Stage 6, just before the PMCs enter meiosis, *BIM1* and *SPL8* displayed a very similar expression domain largely covering the PMCs, their surrounding anther cell wall layers and the carpel marginal meristems of the gynoecium (Figure 5c,f). These data indicate that *BIM1* and *SPL8* expression largely overlap in the early anther and provide further evidence that these genes might act together in regulating anther development.

### 2.5. Both SPL8 and BIM1 Are Conserved in Dicot and Monocot Plants

The anther is an evolutionary well-conserved organ in flowering plants and its complexity and critical importance for fertility imply that the basic molecular genetic mechanisms underlying its development are also conserved. 

**Figure 6:**
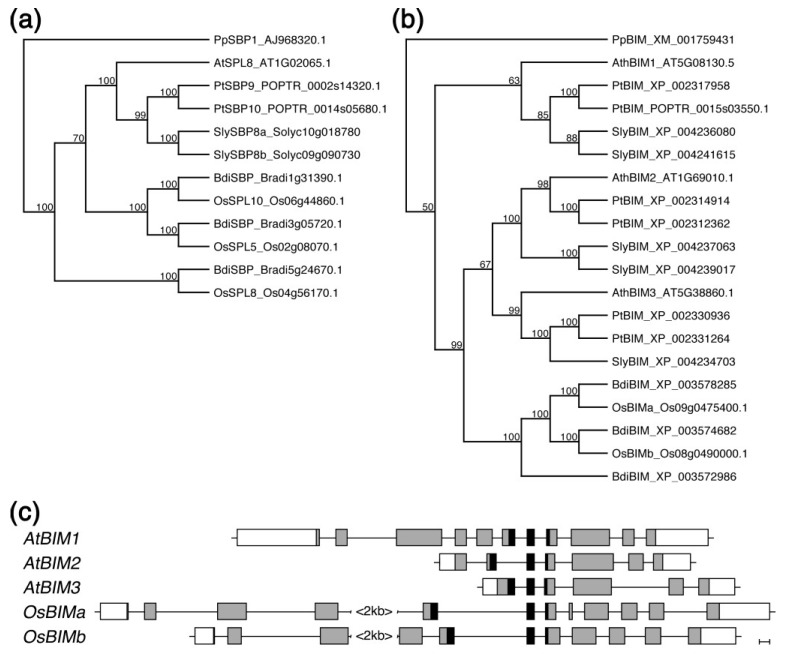
Phylogenetic analysis of SPL8- (**a**) and BIM-like (**b**) proteins. The entire protein sequences encoded by Arabidopsis (AtSPL8, AtBIM), poplar (PtSBP, PtBIM), tomato (SlySBP, SlyBIM), rice (OsSPL, OsBIM), *Brachypodium* (BdiSBP, BdiBIM) and moss (PpSBP, PpBIM) genes were aligned using ClustalW. The phylogram was constructed using the neighbor-joining algorithm using *Physcomitrella patens*-related sequences for rooting. Bootstrap values >50% are shown adjacent to the respective nodes. Database accession numbers are shown after each protein. The genomic organization of the Arabidopsis *BIM1*, *BIM2* and *BIM3* loci are compared to those of the two most closely related *BIM* genes in rice (**c**).

Both SPL8 and BIM1 represent evolutionary diversified and ancient families of transcription factors in plants [17,18,19]. To determine whether other dicots as well as monocots possess highly similar proteins with probably functional homologous properties, we performed a BLAST search of the NCBI GenBank database. In addition, we queried the Arabidopsis SPL8 and BIM1 proteins against the Plant Transcription Factor Database for predicted orthologs (PlantTFDB, [20]). Representing species with available whole genome sequence data, we chose the dicots poplar (*Populus trichocarpa*) and tomato (*Solanum lycopersicum*), and the monocots rice (*Oryza sativa* var. *japonica*) and *Brachypodium distachyon*. Multiple alignments and phylogenetic analysis of the sequences obtained, showed the unique Arabidopsis *SPL8* gene to be represented by two closely related and probably paralogous genes in each of the other species (Figure 6a). Arabidopsis *BIM1* also appeared to be represented by two copies in both other dicot species, as were the related Arabidopsis *BIM2* and *BIM3* genes, with the exception of only one *BIM3*-like copy being identified in tomato (Figure 6b). Interestingly, the BIM-related monocot proteins clustered within a separate group and appeared to be somewhat more related to BIM2 and BIM3. However, a more direct comparison of the monocot proteins suggested an overall higher structural similarity to BIM1, with an extended *N*-terminal domain that was lacking in BIM2 and BIM3. Comparing the respective genomic loci further supported the idea that the identified *BIM*-related monocot genes and Arabidopsis *BIM1* probably share a more ancestral intron-exon structure (Figure 6c).

In addition to Arabidopsis, evolutionary diverged flowering plants express proteins highly similar to SPL8 and BIM1, suggesting a role for these proteins in anther development in other dicots and possibly also in monocots.

## 3. Discussion

In our growth conditions, generally only the first few flowers formed in *spl8* mutant primary inflorescences showed strongly reduced seed set. However, the number of flowers with severely affected fertility dramatically increased in the primary inflorescence of the *spl8 bim1* double mutant (Figure 2). Our results showed that the *bim1* mutation enhanced the *spl8* mutant phenotype with respect to anther histogenesis, suggesting that *BIM1*, similar to *SPL8*, regulates early anther development. Furthermore, *in situ* hybridization revealed a large degree of overlap in the temporal and spatial expression patterns of both genes during early stages of anther development, implying a possible interaction in controlling sporogenous cell formation and the establishment of parietal cell layers in anther patterning.

BIM1 has been identified as a putative SPL8-interacting protein in a yeast two-hybrid screen using SPL8 as bait [21]. It thus appears possible that SPL8 and BIM1, both known to be DNA-binding transcription factors, form a complex to regulate shared target genes in early anther development. This raises the interesting question of whether *SPL8* or other SBP-box genes are involved in brassinosteroid (BR) signaling in controlling anther development. A previous study has shown that BES1 (bri1-Ethylmethane Sulphonate suppressor 1), a key component of the BR signaling pathway, interacts with BIM1 to regulate BR-responsive genes [15]. Another study indicates that BES1 directly binds to the promoter regions of several key genes in anther and pollen development [14]. Therefore, it is tempting to speculate that SPL8 might regulate early anther development as a component of a BES1-BIM1-SPL8 complex. Other SBP-box genes such as miR156-targeted *SPLs*, with functional redundancy to *SPL8* [12], might also join the same pathway to regulate early anther development. The generation and analysis of double mutants between *spl8* and other mutants in BR biosynthesis and signaling will help to clarify whether BIM1 indeed provides a unique link between *SPL8* and the BR pathway.

Our preliminary phylogenetic analysis showed that closely related and putative orthologs of *SPL8* and *BIM1* are identifiable in both monocotyledonous and dicotyledonous plants (Figure 6). However, whereas dicots also appear to possess clear *BIM2* and *BIM3* counterparts, monocot *BIM*-like genes appear to form a separate clade. We showed that in combination with SPL8, BIM1 but not BIM2 or BIM3 affected male fertility. A major difference between these three closely related BIM-like proteins is the presence of a *N*-terminal extension to the bHLH region in BIM1. Differences also exist in the intron-exon structures of the respective genes. Interestingly, this extension is conserved in the monocot BIM sub-family members. Taken together, and in the context of the evolutionary conserved process of anther development, this might imply that a possible interaction between functional SPL8 and BIM1 homologs is expected to be conserved in both mono- and dicotyledonous plants. 

In addition to BR, the phytohormone auxin has also been demonstrated to play a role in anther dehiscence, pollen maturation and filament elongation [22]. Auxin and BR act synergistically, for instance via a BR-mediated degradation of repressors of some auxin activated genes [23]. However, to date there is little support for auxin functioning in early anther development, although some auxin biosynthesis genes are expressed in early anthers [24]. Auxin is known to play an important role in leaf initiation and development and thus probably also in the early development of leaf-derived organs such as anthers and carpels. Moreover, BIM1 interacts with DRN/DRNL, acting directly downstream of auxin responses and is involved in embryonic patterning and lateral organ formation [25,26]. Our own recent data indicate that SBP-box genes such as *SPL8* affect gynoecium development most probably via a link with auxin (Xing and Huijser, unpublished data). Auxin and BR signaling pathways controlling anther development, might thus cross-talk through both *SPL8* and *BIM1* genes.

## 4. Experimental Section

### 4.1. Plant Materials and Growth Conditions

*Arabidopsis thaliana* ecotype Columbia (Col-0; NASC N1092) was used as wild type and all mutants were in the Col-0 background. The mutant alleles *bim1*-1 (Salk_085924, [15]), *bim1*-2 (Salk_132178, [16], *bim2*-1 (Salk_074689, [15] and *bim3*-1 (Salk_079683, [15] and *spl8* single mutants [11] have been described previously. Seeds for *bim1*-3 (N850504) were obtained from the Nottingham Arabidopsis Stock Centre. For identifying homozygouss of *bim1*-3 mutants, a T-DNA Left border primer (5'-AACGTCCGCAATGTGTTATTAAGTTGTC-3'), and two gene-specific primers (5'-TTGCAGCTCGTACGCCGCTGCATCAAGC-3' and 5'-TCTTCCTGTGAACTTCCTTTTGCTGACC-3') were used. Prior to sowing, seeds were imbibed and stratified for 2 days in the dark at 4 °C. Plants were cultivated on prefertilized soil mixture (Type ED73; Werkverband) and grown in the greenhouse at 21–23 °C under long-day conditions (16 h light). 

### 4.2. Histology and Microscopy

Embedding and sectioning of flowers as well as seed set determination were performed according to Xing *et al.* [12]. 

### 4.3. Semi-Quantitative RT-PCR

Inflorescence tips without open flowers of wild type and the *bim1-3* mutant were harvested from 30-day-old plants. RNA isolation and semi-quantitative RT-PCR were performed as described by Xing and Zachgo [10]. The position of the two primers for amplifying part of *BIM1* cDNA transcripts is shown in Figure 3a (P413: 5'-TGTCACCACCAATGATGTTCAATGC-3' and P414: 5'-TCATCTTCCTGTGAACTTCCTTTTGC-3').

### 4.4. *In Situ* RNA Hybridization

RNA *in situ* hybridization was performed as described previously [27]. A *SPL8* antisense probe was prepared according to the method described by Xing *et al.* [12]. To generate *BIM1* antisense probes and to avoid cross-hybridization with other *BIM* gene transcripts, the cDNA sequences of a 220 bp 5' end fragment with primers (5'-GTAGTCACTTGAAAACCCATGATTTTC-3' and 5'-GCTCgtaatacgactcactatagggcTGGTGGTGACAACTCCGGCTTAG-3') and a 540 bp 3' end fragment with primers (5'-GATCATGAAGTTCGTGAACCGGTTTCTCG-3' and 5'-GCTCgtaatacgactcactatagggcTCGTTC ATGCGCTTTGGTTTAG-3'), were amplified as templates, respectively. T7 RNA polymerase (Roche Diagnostics GmbH, Mannheim, Germany) was used for *in vitro* transcription.

### 4.5. Phylogenetic Tree Construction

Multiple alignments of amino acid sequences were generated by the program ClustalW of the MacVector 9.5.2 software package (Accelrys Ltd., Cambridge, UK) using the Gonnet matrix with an open gap penalty of 10 and an extend gap penalty of 0.1. The phylograms based on these alignments were constructed using the neighbor-joining algorithm available in the same software package. A bootstrap analysis with 10,000 repetitions was performed with random tiebreaking and gaps in the multiple alignments ignored.

## 5. Conclusions

Based on the results in this study, we conclude that BIM1 provides a newly identified early anther gene function together with SPL8 in the same or in a parallel pathway to control anther development by regulating sporogenous cell formation and the differentiation and patterning of anther wall cell layers. In addition, BIM1 is a component of the brassinosteroid signaling pathway. Thus, *SPL8* and other miR156-targeted *SPL* genes might join the BR signaling via a link with BIM1 in regulating the early events of anther development.

## References

[1] Coen E.S., Meyerowitz E.M. (1991). The war of the whorls: Genetic interactions controlling flower development. Nature.

[2] Bowman J.L., Baum S.F., Eshed Y., Putterill J., Alvarez J. (1999). Molecular genetics of gynoecium development in Arabidopsis. Curr. Top. Dev. Biol..

[3] Scott R.J., Spielman M., Dickinson H.G. (2004). Stamen structure and function. Plant Cell.

[4] Causier B., Schwarz-Sommer Z., Davies B. (2010). Floral organ identity: 20 years of ABCs. Semin. Cell Dev. Biol..

[5] Sanders P.M., Bui A.Q., Weterings K., McIntire K.N., Hsu Y.C., Lee P.Y., Truong M.T., Beals T.P., Goldberg R.B. (1999). Anther developmental defects in *Arabidopsis thaliana* male-sterile mutants. Sex. Plant Reprod..

[6] Ma H. (2005). Molecular genetic analyses of microsporogenesis and microgametogenesis in flowering plants. Annu. Rev. Plant Biol..

[7] Xing S., Salinas M., Huijser P. (2011). New players unveiled in early anther development. Plant Signal. Behav..

[8] Yang W.C., Ye D., Xu J., Sundaresan V. (1999). The *SPOROCYTELESS* gene of *Arabidopsis* is required for initiation of sporogenesis and encodes a novel nuclear protein. Genes Dev..

[9] Schiefthaler U., Balasubramanian S., Sieber P., Chevalier D., Wisman E., Schneitz K. (1999). Molecular analysis of *NOZZLE*, a gene involved in pattern formation and early sporogenesis during sex organ development in *Arabidopsis thaliana*. Proc. Natl. Acad. Sci. USA.

[10] Xing S., Zachgo S. (2008). *ROXY1* and *ROXY2*, two *Arabidopsis* glutaredoxin genes, are required for anther development. Plant J..

[11] Unte U.S., Sorensen A.M., Pesaresi P., Gandikota M., Leister D., Saedler H., Huijser P. (2003). *SPL8*, an SBP-box gene that affects pollen sac development in *Arabidopsis*. Plant Cell.

[12] Xing S., Salinas M., Höhmann S., Berndtgen R., Huijser P. (2010). miR156-targeted and nontargeted SBP-box transcription factors act in concert to secure male fertility in *Arabidopsis*. Plant Cell.

[13] Kutschera U., Wang Z.Y. (2012). Brassinosteroid action in flowering plants: A darwinian perspective. J. Exp. Bot..

[14] Ye Q., Zhu W., Li L., Zhang S., Yin Y., Ma H., Wang X. (2010). Brassinosteroids control male fertility by regulating the expression of key genes involved in *Arabidopsis* anther and pollen development. Proc. Natl. Acad. Sci. USA.

[15] Yin Y., Vafeados D., Tao Y., Yoshida S., Asami T., Chory J. (2005). A new class of transcription factors mediates brassinosteroid-regulated gene expression in *Arabidopsis*. Cell.

[16] Chandler J.W., Cole M., Flier A., Werr W. (2009). BIM1, a bHLH protein involved in brassinosteroid signalling, controls *Arabidopsis* embryonic patterning via interaction with DORNRÖSCHEN and DORNRÖSCHEN-LIKE. Plant Mol. Biol..

[17] Riese M., Höhmann S., Saedler H., Münster T., Huijser P. (2007). Comparative analysis of the SBP-Box gene families in *P. patens* and seed plants. Gene.

[18] Salinas M., Xing S., Höhmann S., Berndtgen R., Huijser P. (2012). Genomic organization, phylogenetic comparison and differential expression of the SBP-box family of transcription factors in tomato. Planta.

[19] Pires N., Dolan L. (2010). Origin and diversification of basic-helix-loop-helix proteins in plants. Mol. Biol. Evol..

[20] Zhang H., Jin J.P., Tang L., Zhao Y., Gu X.C., Gao G., Luo J.C. (2011). PlantTFDB 2.0: update and improvement of the comprehensive plant transcription factor database. Nucleic Acids Res..

[21] Zhang Y. (2005). The SBP-box gene *SPL8* affects reproductive development and gibberellin response in Arabidopsis. Ph.D. Thesis.

[22] Cecchetti V., Altamur M.M., Falasca G., Costantino P., Cardarelli M. (2008). Auxin regulates Arabidopsis anther dehiscence, pollen maturation, and filament elongation. Plant Cell.

[23] Vert G., Walcher C.L., Chory J., Nemhauser J.L. (2008). Integration of auxin and brassinosteroid pathways by Auxin Response Factor 2. Proc. Natl. Acad. Sci. USA.

[24] Sundberg E., Østergaard L. (2009). Distinct and dynamic auxin activities during reproductive development. Cold Spring Harb. Perspect. Biol..

[25] Kirch T., Simon R., Grünewald M., Werr W. (2003). The *DORNRÖSCHEN/ENHANCER OF SHOOT REGENERATION1* gene of *Arabidopsis* acts in the control of meristem cell fate and lateral organ development. Plant Cell.

[26] Chandler J.W., Cole M., Flier A., Grewe B., Werr W. (2007). The AP2 transcription factors DORNRÖSCHEN and DORNRÖSCHEN-LIKE redundantly control Arabidopsis embryo patterning via interaction with PHAVOLUTA. Development.

[27] Xing S., Rosso M.G., Zachgo S. (2005). ROXY1, a member of the plant glutaredoxin family, is required for petal development in *Arabidopsis thaliana*. Development.

